# Acute Physiological Responses to Prolonged Sedentary Behavior: Impact on Cardiovascular Function and Muscle Activity in Young Adults

**DOI:** 10.3390/jfmk11010041

**Published:** 2026-01-19

**Authors:** Jonas Ribeiro Gomes da Silva, Antônio Ribeiro Neto, Dernival Bertoncello, Jeffer Eidi Sasaki, Moacir Marocolo, Nicolas Bueno Alves, Sheilla Tribess, Ciro José Brito, Jair Sindra Virtuoso Junior

**Affiliations:** 1 Graduate Program in Health Care, Institute of Health Sciences, Federal University of Triângulo Mineiro, Uberaba 38025-180, Brazil; jonasrgs.contato@gmail.com (J.R.G.d.S.); antoniorn11@yahoo.com.br (A.R.N.); 2 Department of Applied Physiotherapy, Institute of Health Sciences, Federal University of Triângulo Mineiro, Uberaba 38025-180, Brazil; dernival.bertoncello@uftm.edu.br; 3 Department of Kinesiology, University of Wisconsin-Madison, Madison, WI 53706, USA; jesasaki@wisc.edu; 4 Department of Biophysics and Physiology, Institute of Biological Sciences, Federal University of Juiz de Fora, Juiz de Fora 36036-900, Brazil; isamjf@gmail.com; 5 Department of Sport Sciences, Institute of Health Sciences, Federal University of Triângulo Mineiro, Uberaba 38025-180, Brazil; bueno11nicolas@gmail.com (N.B.A.); sheilla.tribess@uftm.edu.br (S.T.); 6 Department of Physical Education, Institute of Life Sciences, Federal University of Juiz de Fora, Juiz de Fora 36036-900, Brazil

**Keywords:** electromyography, hemodynamics, postural physiology, sedentary behavior, vascular response

## Abstract

**Background:** Prolonged sitting has been associated with adverse cardiovascular and neuromuscular responses; however, the temporal onset of these acute physiological changes remains unclear. This study aimed to determine the acute effects of prolonged sitting on blood flow, blood pressure, and muscle activity. **Methods:** A non-controlled clinical trial was conducted with 21 healthy adults (22.5 ± 1.60 years), both male and female. Participants remained seated continuously for three hours, with data collected every 20 min, including infrared thermography, blood pressure, and electromyographic activity. Skin temperature was measured using infrared thermography on the calf region of both legs, and the mean temperature was analyzed. Systolic and diastolic blood pressure were measured using an oscillometric device, and mean arterial pressure was subsequently calculated. Muscle activity was assessed through surface electromyography, using median frequency and root mean square values. Statistical analysis was performed using the Friedman test and the Durbin–Conover post hoc test, along with a subjective trend analysis of each variable over time. **Results:** A significant reduction was observed in both calf skin temperature and median frequency after 60 min of uninterrupted sitting (*p* < 0.05). Mean and systolic blood pressure exhibited an increasing trend after 160 min (*p* < 0.05). **Conclusions:** The exposure–response data from this study may contribute to the planning of future interventions aimed at refining recommendations for breaking up prolonged sitting periods.

## 1. Introduction

Sedentary Behavior (SB) is defined as activities performed while sitting, reclining, or lying down, with energy expenditure below 1.5 metabolic equivalents during waking hours [1]. Growing evidence suggests that prolonged SB is associated with adverse physiological effects, including elevated blood pressure (BP), impaired biomarkers, and vascular dysfunction [2,3,4,5].

The vascular dysfunction observed in the lower limbs during uninterrupted sitting is primarily attributed to a reduction in shear stress resulting from decreased blood flow [6]. This decline triggers harmful changes in BP regulation mechanisms, increasing the risk of hypertension [7]. Additionally, the marked reduction in muscle activity while seated eliminates the action of the muscle pump, which is critical for venous return and circulatory homeostasis [6]. Metabolic and vascular alterations seem to occur within as little as three hours of uninterrupted SB [4,8]. Acute changes in blood flow have been detected after just 30 min of sitting, indicating early signs of vascular dysfunction [4,9,10]. To mitigate these effects, breaking up prolonged sitting with physical activity bouts has been shown to improve vascular parameters and reduce cardiovascular risk [2,3,11].

Despite these findings, the exact moment at which these physiological changes begin remains unclear. Current intervention protocols largely rely on arbitrary intervals for interrupting SB time, typically ranging from every 20 min to every two hours, primarily due to the lack of evidence identifying the precise timing at which hemodynamic and neuromuscular changes begin [11]. This gap limits the development of evidence-based recommendations for health professionals and public health guidelines.

More precise evidence in this field will support the development of more effective interventions, with clearer thresholds for interrupting SB. Accordingly, the findings of this study may provide a basis for recommendations that adopt better-defined dosing strategies, moving beyond the generic advice to “sit less and move more”. The present study aims to identify the onset of acute alterations in blood pressure, blood flow, and muscle activity induced by uninterrupted sitting.

## 2. Materials and Methods

### 2.1. Study Design

This study was designed as a non-controlled clinical trial with a prospective approach, aiming to determine the minimum duration of prolonged SB required to elicit acute alterations in cardiovascular parameters and muscle activity in young adults. The study was registered in the Brazilian Clinical Trials Registry (REBEC) (RBR-32dp35h) and adhered to the Transparent Reporting of Evaluations with Nonrandomized Designs (TREND) statement [12]. The research protocol was approved by the Local Research Ethics Committee for Human Studies (approval number 6.209.646). All participants signed a written informed consent form prior to enrollment, agreeing to voluntarily participate in the study.

Participants were eligible if they were healthy young adults aged 18–24 years, with no diagnosed chronic diseases, not using continuous medication and/or any ergogenic aids, and without any injuries or conditions that would prevent prolonged sitting. Failure to complete any phase of the study, including initial screening, assessments, or the experimental session, was considered a criterion for exclusion.

A priori sample size calculation was conducted using G*Power 3.1.9.6, considering the study design (single-group), the most appropriate statistical test (one-way ANOVA), a statistical power of 0.80, and pilot study data on the number of measurements to be performed. Based on an effect size of 0.25 and a correlation coefficient of 0.50 between repeated measurements, the required sample size was estimated to be 14 participants.

### 2.2. Experimental Protocol

Participants were instructed to avoid prolonged sun exposure for at least five days before the experimental protocol (to minimize the risk of sunburn), hot showers within two hours of the session, and the application of creams or ointments on the skin [13].

Additionally, they were required to refrain from moderate-to-vigorous physical activity, caffeine and alcohol consumption for 24 h before the experimental session while maintaining their usual dietary and sleep habits.

**Figure 1:**
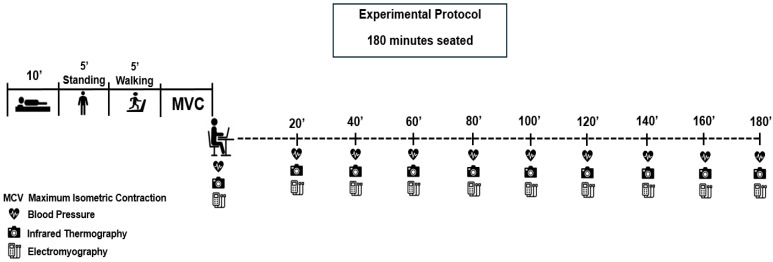
Experimental design of the study.

All experimental sessions were conducted at the Laboratory between 7:00–11:00 a.m. In addition to sample characterization at both the beginning and end of the study, the experimental session was structured into two distinct phases: (i) Pre-experimental protocol phase, which included the collection of baseline and reference measurements. (ii) Experimental protocol phase, in which participants remained in prolonged sedentary behavior for three hours (Figure 1).

Upon arrival at the laboratory, participants underwent a comprehensive anthropometric assessment, including measurements of body mass (kg), height (m), waist circumference (cm), hip circumference (cm), and body composition analysis. From these measurements, the body mass index (BMI, kg/m^2^) and waist-to-hip ratio were calculated.

Subsequently, participants received individualized instructions at three different time points regarding the electromyography (EMG) data collection procedures, which were used for signal normalization. After the anthropometric evaluation, participants lay supine on a medical examination table for 10 min to achieve a steady-state condition. This period also allowed for proper skin preparation and electrode placement for EMG recording, as well as acclimatization to the experimental environment. Next, participants stood in an upright position for five minutes, as this is considered the first posture outside of SB [1]. They then performed a five-minute treadmill walk at a speed of 3.5 km/h, with the exercise intensity standardized as “light” based on the Rating of Perceived Exertion scale [14].

Finally, they underwent a maximal voluntary isometric contraction (MVIC) test of the medial gastrocnemius muscle of the right lower limb, which was used for EMG signal normalization.

During this period, participants were allowed minor free movements of their upper and lower limbs, such as adjusting posture or handling personal devices, but without engaging in any vigorous or physically demanding activity. However, interruptions of the experimental session were not permitted. If an interruption occurred for any reason unrelated to the protocol, the session was canceled and rescheduled to ensure the standardization of experimental conditions.

From this point onward, participants assumed the seated position, and data collection for all investigated variables was then performed at 20-min intervals, with the first measurement taken immediately after they were seated. EMG data were continuously recorded for five minutes during each collection period. To ensure protocol standardization, the intervals between collections were adjusted according to the duration of EMG recording: the first and second measurements were separated by 12.5 min, while subsequent measurements were taken at 15-min intervals. This adjustment was necessary to align data collection sequences with the total duration of the experimental session.

### 2.3. Physical Activity Level

Participant’s physical activity level was assessed using the 24-Hour Physical Activity Recall (R24AF) [15]. Data collection was conducted by a trained researcher through a structured interview, guiding participants to recall in detail their daily activity routine over a typical day in the previous week.

The information provided by the participants included a detailed description of the type of activity performed, encompassing its nature, mode of execution, and duration [15].

Following data collection, the Metabolic Equivalent of Task (MET) for each reported activity was assigned based on the Compendium of Physical Activities [16]. Activities were then classified according to their intensity (light, moderate, or vigorous), and the total time spent in SB was determined [17]. Additionally, the total duration (in minutes) dedicated to each activity during the participant’s waking hours on a typical day was calculated.

### 2.4. Blood Pressure Measurement

Systolic blood pressure (SBP) and diastolic blood pressure (DBP) were measured using a validated oscillometric device (Omron^®^ HEM-780-E, Omron Healthcare Co., Ltd., Kyoto, Japan). Three measures were taken with a one-minute interval between each reading, always on the participant’s left arm. For analysis, the mean of the last two measurements was considered, following standardization protocols [18].

Following the acquisition of SBP and DBP values, mean arterial pressure (MAP) was calculated. MAP represents the weighted average of arterial pressure throughout the cardiac cycle, incorporating both systolic and diastolic phases. It is determined by cardiac output and systemic vascular resistance, both of which are modulated by various physiological factors [19,20]. The MAP calculation followed the widely accepted formula in the literature: MAP = (1/3 * SBP) + (2/3 * DBP) [19,20].

### 2.5. Blood Flow Assessment

**Figure 2:**
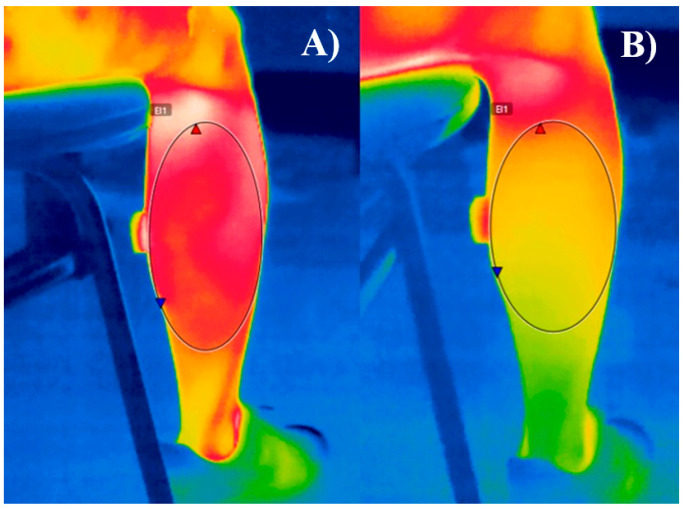
Gastrocnemius area assessed before (**A**) and after (**B**) three hours of sitting time. It can be depicted that (**A**) shows a higher thermal gradient and skin temperature compared to (**B**), indicating a potential reduction in local blood flow.

Lower limb blood flow was evaluated using infrared thermography, a non-invasive analysis tool widely employed for monitoring physiological functions associated with skin temperature regulation [21]. This method enables the detection of infrared radiation emitted by the body, allowing the identification of thermal variations that reflect changes in blood flow [21] (Figure 2).

To acquire thermographic images, a thermal camera (FLIR^®^ E76, FLIR Systems, Inc., Wilsonville, OR, USA) with a resolution of 320 × 240 pixels was used. The equipment was installed at the data collection site 60 min before measurements to ensure proper environmental acclimatization. Skin emissivity was standardized at ε = 0.98 [12]. Infrared thermography is a validated technique for blood flow assessment, demonstrating correlation with Doppler ultrasound, which is considered the gold standard for endothelial function measurement. Scientific evidence indicates that reductions in skin temperature are associated with decreased blood flow, reinforcing the applicability of this method for peripheral perfusion analysis [22,23].

### 2.6. Electromyographic Activity Assessment

Electromyographic (EMG) activity was assessed using surface EMG with a DELSYS^®^ system (DSY-DS-T01-8, Delsys, Inc., Natick, MA, USA). EMG signals were acquired through the TRIGNO^®^ software (version 2.0.1.3, Delsys Inc., Natick, MA, USA), employing a sampling rate of 2000 Hz for each recording [24].

Electrode placement followed the guidelines outlined in the SENIAM (Surface Electromyography for Non-Invasive Assessment of Muscles) protocol [25]. To optimize signal acquisition and minimize impedance, standardized skin preparation procedures—including cleansing and abrasion—were performed prior to electrode placement. The electrodes were aligned parallel to the muscle fibers of the gastrocnemius medialis (GMD).

During the protocol involving prolonged exposure to SB, EMG activity was recorded in 5-min intervals, with a 15-min break between successive recordings, ensuring an average interval of 20 min between assessments. To standardize data collection, participants were informed in advance of the start and end times of each EMG recording. Throughout data acquisition, participants were instructed to keep their right leg as still as possible during the 5-min recording period.

Following data acquisition, signal processing was conducted in accordance with the methodology proposed by Oliveira et al. [23] to extract Root Mean Square (RMS) and Median Frequency values. Data processing was performed using Matlab^®^ (The MathWorks Inc., Natick, MA, USA) and Octave (GNU), ensuring data integrity and eliminating potential external interferences that could compromise EMG signal quality.

For the maximal voluntary isometric contraction (MVIC) of the GMD, participants were positioned in a chair identical to that used in the SB exposure protocol, maintaining 90° of hip flexion with the knee fully extended and supported, ensuring that only the ankle remained free. This configuration minimized compensatory movements and prevented electrode displacement. The participants’ arms were stabilized at shoulder level, and two researchers ensured postural fixation by keeping the chair static throughout the test. Additionally, a portable device was placed against the participant’s foot, allowing them to apply force against a fixed surface to ensure proper execution of the isometric contraction.

During the MVIC test, participants received continuous verbal encouragement to maximize plantar flexion effort. Data were collected over three trials, each lasting 5 s, with 1-min rest intervals between trials. For subsequent analysis, the mean of the three attempts was calculated.

### 2.7. Statistical Analysis

Descriptive statistics were used to present the data, with results expressed as mean and standard deviation for continuous variables and absolute frequency distribution for categorical variables. The normality of data distribution was assessed using the Shapiro–Wilk test. For variables measured at multiple time points throughout the SB exposure period, the Friedman test was applied to determine whether significant differences existed over time. When significant differences were detected, post hoc analysis was conducted using the Durbin–Conover test to identify specific time points where changes occurred. The correlation between variables was analyzed using Spearman’s correlation coefficient, considering changes between minute 180 and baseline (minute 0). All statistical analyses were conducted with a significant level of 5%, using JAMOVI 2.4.8.

## 3. Results

### 3.1. Sample and Hemodynamic Measurements

A total of 21 young adults (11 men and 10 women) participated in the study. The general characteristics of the sample are presented in Table 1.

**Figure 3:**
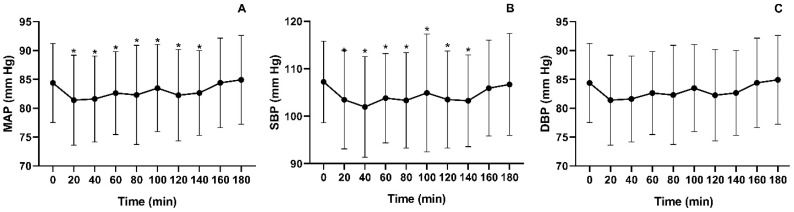
Mean values of (**A**) MAP, (**B**) SBP, and (**C**) DBP over three hours of prolonged sedentary behavior. Significant difference compared to minute 0 (* *p* < 0.05).

Statistical analysis using the Friedman test revealed significant changes in MAP over time [χ^2^(9) = 24.274; *p* < 0.004]. A transient peak in MAP was observed around minute 60; however, statistically significant differences compared to baseline (84.4 ± 6.84 mmHg) were only detected from minute 160 (84.4 ± 7.76 mmHg) and minute 180 (84.9 ± 7.69 mmHg) onward (Figure 3A). Similarly, SBP fluctuated over time, as indicated by the Friedman test [χ^2^(9) = 26.778; *p* < 0.002]. A peak in SBP was noted around minute 60, but significant differences compared to baseline (107 ± 8.57 mmHg) were only found at minute 160 (106 ± 10.1 mmHg) and minute 180 (107 ± 10.7 mmHg) (Figure 3B). For DBP, a peak was observed at minute 40; however, statistical analysis did not reveal any significant differences between time points [χ^2^(9) = 14.578; *p* = 0.103] (Figure 3C).

### 3.2. Skin Temperature Assessment

**Figure 4:**
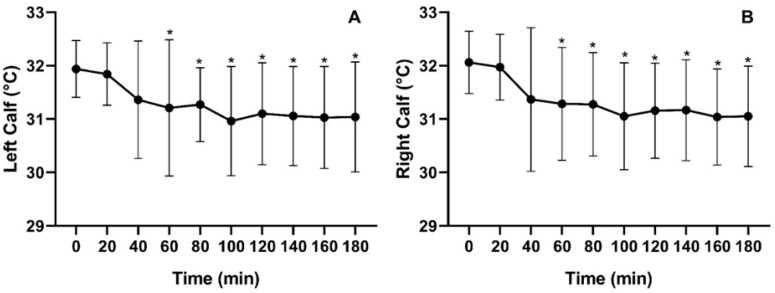
Mean skin temperature of the left (**A**) and right (**B**) calves measured during three hours of prolonged SB. * *p* < 0.05.

Mean skin temperature varied significantly over time for both the left calf [χ^2^(9) = 71.687; *p* < 0.000] and the right calf [χ^2^(9) = 75.292; *p* < 0.000]. Throughout the three-hour period of uninterrupted SB, an average temperature decrease of −0.90 ± 1.11 °C was observed for the left calf, while the right calf exhibited a decline of −1.01 ± 9.96 °C. Despite a downward trend starting at minute 20, statistically significant reductions compared to baseline were only detected from minute 60 onward for both calves (Figure 4).

### 3.3. Muscle Activation Analysis

**Figure 5:**
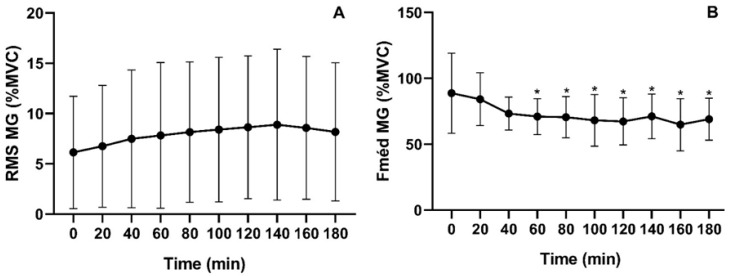
Electromyographic parameters of the medial gastrocnemius during prolonged SB. RMS = Root mean square activation (%) relative to maximal voluntary isometric contraction (MVIC) (**A**); Median frequency (Fmed) relative to MVIC (**B**). * *p* < 0.05.

Figure 5 illustrates the percentage of activation over time during prolonged SB for the median frequency (Fmed) and root mean square (RMS) relative to the maximal voluntary isometric contraction (MVIC) of the gastrocnemius medialis (GMD). For Fmed, the mean activation of the GMD was 72.89% ± 7.61, with statistically significant differences compared to baseline detected from minute 60 onward [χ^2^(9) = 42; *p* < 0.001]. In contrast, RMS activation averaged 7.91% ± 0.88, with no significant differences observed between time points [χ^2^(9) = 7.18; *p* = 0.619].

### 3.4. Correlation Analysis

Spearman’s rank correlation coefficient did not reveal any statistically significant associations between the variables analyzed (*p* > 0.05 for all comparisons). No meaningful relationships were found between changes in muscle activation, skin temperature, or cardiovascular parameters during the sitting protocol.

## 4. Discussion

The objective of this study was to analyze the acute effects of prolonged SB on blood pressure, blood flow, and muscle activity in young adults. The results indicate that, for MAP and BP, no significant differences were observed at 160 min of prolonged sitting compared to baseline. However, skin temperature showed a significant decrease in both calves starting at 60 min, while MeanFreq exhibited a significant reduction from 80 min onward.

During prolonged sitting, skin temperature was the first physiological parameter to exhibit significant changes, decreasing markedly after one hour. One of the primary factors regulating skin temperature is blood flow, and a decline in skin temperature is generally associated with reduced blood flow to the assessed region [21,22,23]. A temperature variation greater than 1 °C within the same body region over time can indicate an underlying pathophysiological process [21]. In this study, we observed a decrease of −0.90 °C in the left calf and −1.01 °C in the right calf after three hours, suggesting a potential deleterious effect on endothelial and vascular health when chronically exposed to prolonged sitting. Uninterrupted sitting for prolonged periods, particularly between 120 and 180 min, has been linked to a progressive decline in lower limb vascular function, with more pronounced impairments as sitting duration increases [2,4].

While previous studies did not identify significant vascular function changes within shorter timeframes (e.g., 30 to 60 min) [2,4], evidence suggests that extended exposure to prolonged sitting may negatively impact endothelial function and hemodynamic regulation even in young, healthy individuals [4].

The findings of the present study partially align with this evidence, as vascular function alterations were observed after 180 min of prolonged sitting. However, unlike prior research, significant changes were detected as early as 60 min, with a downward trend in skin temperature emerging from 20 min. These findings suggest that vascular impairments may initiate earlier than previously thought, potentially affecting peripheral circulation from the initial minutes of sitting.

One possible physiological mechanism underlying these changes is the reduction in venous return from the lower limbs, caused by increased pressure in the posterior thigh region. This pressure leads to arterial constriction and an increase in hydrostatic pressure [6,26]. This effect is exacerbated by reduced muscle activity during prolonged sitting, eliminating the contribution of the calf muscle pump in facilitating venous return [6,27].

The decline in calf muscle activity compromises the efficiency of the muscle pump, leading to increased fluid retention in the lower limbs during extended sitting [28]. The reduction in muscle temperature observed in this context serves as an indirect marker of decreased myoelectric activity, suggesting lower calf muscle activation and impaired peripheral circulation [27,28].

In this study, we observed a decline in MeanFreq of the gastrocnemius medialis, a parameter widely used to assess and monitor muscle fatigue [29,30]. The reduction in MeanFreq, evident from 80 min of prolonged sitting, may indicate neuromuscular fatigue, reflecting decreased contractile efficiency and potential physiological adaptations due to prolonged inactivity. Based on these results, we hypothesize that, similar to isometric contraction exercises, reduced blood flow in a specific region leads to decreased oxygenation and energy substrate availability, ultimately resulting in acute muscle fatigue and impaired muscle contractile mechanisms [31].

Additionally, the literature suggests that prolonged sitting with low lower-limb muscle activity reduces both blood flow and shear stress—key mechanisms for vascular regulation [2,6]. However, our findings indicate a distinct sequence of events, where a reduction in blood flow (detected via infrared thermography) occurs first, followed by decreased muscle activity and, subsequently, an increase in blood pressure. A potential reduction in blood flow may decrease shear rate, directly impacting NO synthesis and availability, an essential regulator of vasodilation [9,10]. A decline in circulating NO compromises vascular wall elasticity, increasing arterial stiffness and elevating blood pressure due to impaired vascular adaptation to fluctuations in cardiac output [9,10,32].

Thus, prolonged sitting leads to increased MAP and SBP but does not significantly affect DBP [3], particularly in young adults. This trend was observed in our study, where MAP and SBP reached values comparable to baseline after 160 min of prolonged sitting. However, we identified an initial peak increase at 60 min, suggesting that early physiological alterations may occur before the timeframes typically reported in literature.

These findings offer valuable insights into the development of targeted interventions. Previous studies have suggested that breaking up sitting time at intervals ranging from 20 to 120 min may mitigate the cardiovascular effects of prolonged sedentary behavior [11]. Although each physiological variable exhibits changes at different time points, our data indicates that early physiological changes may occur between 40 and 60 min, even if statistical significance is not reached within this interval. This study provides an initial contribution to understanding the effects of prolonged sitting time, as most research in this area has focused on evaluating the impact of different interventions on cardiovascular parameters [2,3,11] or comparing muscle activation during sedentary behavior with various daily activities [33,34,35,36,37].

From a practical standpoint, the relevance of the present study lies in its contribution to refining the understanding of the temporal sequence of acute physiological changes induced by SB. This knowledge may support the development of evidence-based strategies for interrupting SB. The identification of early physiological markers, such as reductions in skin temperature and electromyographic activity, suggests the potential for establishing physiological thresholds to guide the optimal timing of movement breaks, which may ultimately strengthen clinical and public health initiatives aimed at reducing SB.

However, some important limitations must be considered when interpreting these findings. The absence of a control group limits the ability to distinguish the specific effects of the experimental protocol from potential circadian or environmental influences, and anticipatory responses may have occurred despite the standardized procedures. In addition, although infrared thermography is a reliable indirect indicator of peripheral perfusion, it does not allow direct assessment of shear stress or endothelial function.

The sample consisted exclusively of healthy young adults, which also restricts the generalizability of the results. Therefore, future studies should prioritize controlled research designs with larger and more heterogeneous samples, as well as longer experimental protocols that simulate a typical working day, comparing conditions with and without interruptions for physical activity. Such investigations may contribute to identifying physiological thresholds that guide the ideal frequency of breaks to interrupt SB.

## 5. Conclusions

This study expands the understanding of the temporal sequence of vascular and neuromuscular responses during uninterrupted sitting in healthy young adults. We demonstrated that reductions in calf skin temperature and electromyographic activity occur before increases in blood pressure, suggesting that circulatory and muscular impairments begin within the first hour of sitting. This chronology provides more precise evidence to guide the frequency of interruption, moving beyond the practice of adopting arbitrary intervals. The identification of early physiological markers of sedentary stress reinforces the need to implement regular active breaks, both in clinical and occupational settings and in sports recovery protocols where prolonged sedentary periods are common. By establishing exposure-response thresholds, these findings contribute to the development of individualized strategies and to the refinement of recommendations aimed at protecting cardiovascular and musculoskeletal health across populations.

## Figures and Tables

**Table 1:** Descriptive characteristics of the study participants.

Parameter	Mean ± SD
Age (Years)	22.5 ± 1.6
Weight (kg)	70.9 ± 15
Height (m)	1.72 ± 0.1
BMI (kg/m^2^)	23.9 ± 3.0
WC (cm)	78.3 ± 9.2
WHR	0.7 ± 0.1
BF (%)	24.4 ± 7.6
MM (kg)	29.3 ± 9.7
FFM (kg)	56 ± 17.5
SB (min)	540 ± 190
LPA (min)	210 ± 78.6
MPA (min)	159 ± 20.1
VPA (min)	23.8 ± 30.4

Values are means ± SD (*n* =21); BMI = Body Index Mass; WC = Waist Circumference; WHR = Waist-to-hip Ratio; BF = Body Fat; MM = Muscle Mass; FFM = Free Fat Mass; SB = Sedentary Behavior; LPA = Low Physical Activity; MPA = Moderate Physical Activity; VPA = Vigorous Physical Activity.

## Data Availability

The data are available for access at the following address: https://figshare.com/s/8de96104c41f7396bd45 (accessed on 9 January 2026).

## Abbreviations

The following abbreviations are used in this manuscript:

BMI	Body Mass Index
BP	Blood Pressure
DBP	Diastolic Blood Pressure
EMG	Electromyography
Fmed	Median Frequency
MAP	Mean Arterial Pressure
MVIC	Maximal Voluntary Isometric Contraction
RMS	Root Mean Square
SB	Sedentary Behavior
SBP	Systolic Blood Pressure
